# Supplementary material for: Multimodal Molecular Imaging Reveals Tissue-Based T Cell Activation and Viral RNA Persistence for Up to 2 Years Following COVID-19
Source: medRxiv. 2023 Jul 31:2023.07.27.23293177. Preprint. [Version 1] doi: 10.1101/2023.07.27.23293177 (PMC10418298; doi:10.1101/2023.07.27.23293177)
Supplement: Supplement 1 [file NIHPP2023.07.27.23293177v1-supplement-1.pdf]

medRxiv preprint doi: https://doi.org/10.1101/2023.07.27.23293177; this version posted July 31, 2023. The copyright holder for this preprint (which was not certified by peer review) is the author/funder, who has granted medRxiv a license to display the preprint in perpetuity.
It is made available under a CC-BY 4.0 International license .

SUPPLEMENTAL INFORMATION

Supplemental Table 1. Clinical Findings on Chest CT Scans

Pt #	Days from Infection to PET Scan[a]	Sex	Age Bracket (Years)	Hosp	LC Symptom Count[b]	Abnormal Findings on Chest CT as Determined by Two Cross-Sectional Radiologists (R1, R2)
1	27	F	25-29	N	2	None
2	29	M	35-39	N	1	R Lower Lobe Bulla (R1); Minimal basilar subpleural bands/reticulation/ground glass (R2)
3	42	M	30-34	N	4	None
4	44	M	60-64	N	0	Minimal subpleural reticulation and banding in bases, L>R (R2)
5	48	M	25-29	N	5	None
6	50	F	30-34	N	4	Few tiny subpleural micronodules, likely intrapulmonary lymph nodes (R2)
7	64	M	40-44	N	14	None
8	65	F	60-64	N	0	Mild apical scarring
9	83	F	55-59	N	0	None
10	95	M	45-49	N	0	Calcified R lower lobe granuloma, L lower lobe subpleural granuloma (R1)
11	114	F	30-34	N	8	Subpleural L lower lobe nodule, intrapulmonary lymph node (R1)
12	193	F	25-29	N	13	None
13	205	F	45-49	N	6	Minimal apical, lingular and R middle lobe consolidation or scarring (R1)
14	231	M	35-39	N	4	None
15	246	M	65-69	N	6	None
16	260	M	50-54	Y[c]	8	R lower lobe nodule <1 centimeter (R1 & R2)
17	406	F	30-34	N	7	L major fissure micronodule, intrapulmonary lymph node (R1)
18	617	F	30-34	N	14	None
19	625	M	45-49	N	11	None
20	641	M	25-29	N	15	None
21	654	F	30-34	N	6	None
22	663	M	50-54	N	0	Mild apical scarring
23	890	M	50-54	N	6	Basilar predominant subpleural reticulation and scarring
24	910	F	55-59	Y[d]	0	None

R = right; L = left; M = male; F = female
[a] Days from last COVID-19 vaccine dose to PET imaging
[b] Number of participant reported symptoms at the time of PET imaging (out of 32 total)
[c] Participant did not require intensive care but received supplemental oxygen during hospitalization
[d] Participant did not require intensive care or supplemental oxygen during hospitalization

medRxiv preprint doi: https://doi.org/10.1101/2023.07.27.23293177; this version posted July 31, 2023. The copyright holder for this preprint
(which was not certified by peer review) is the author/funder, who has granted medRxiv a license to display the preprint in perpetuity.
It is made available under a CC-BY 4.0 International license .

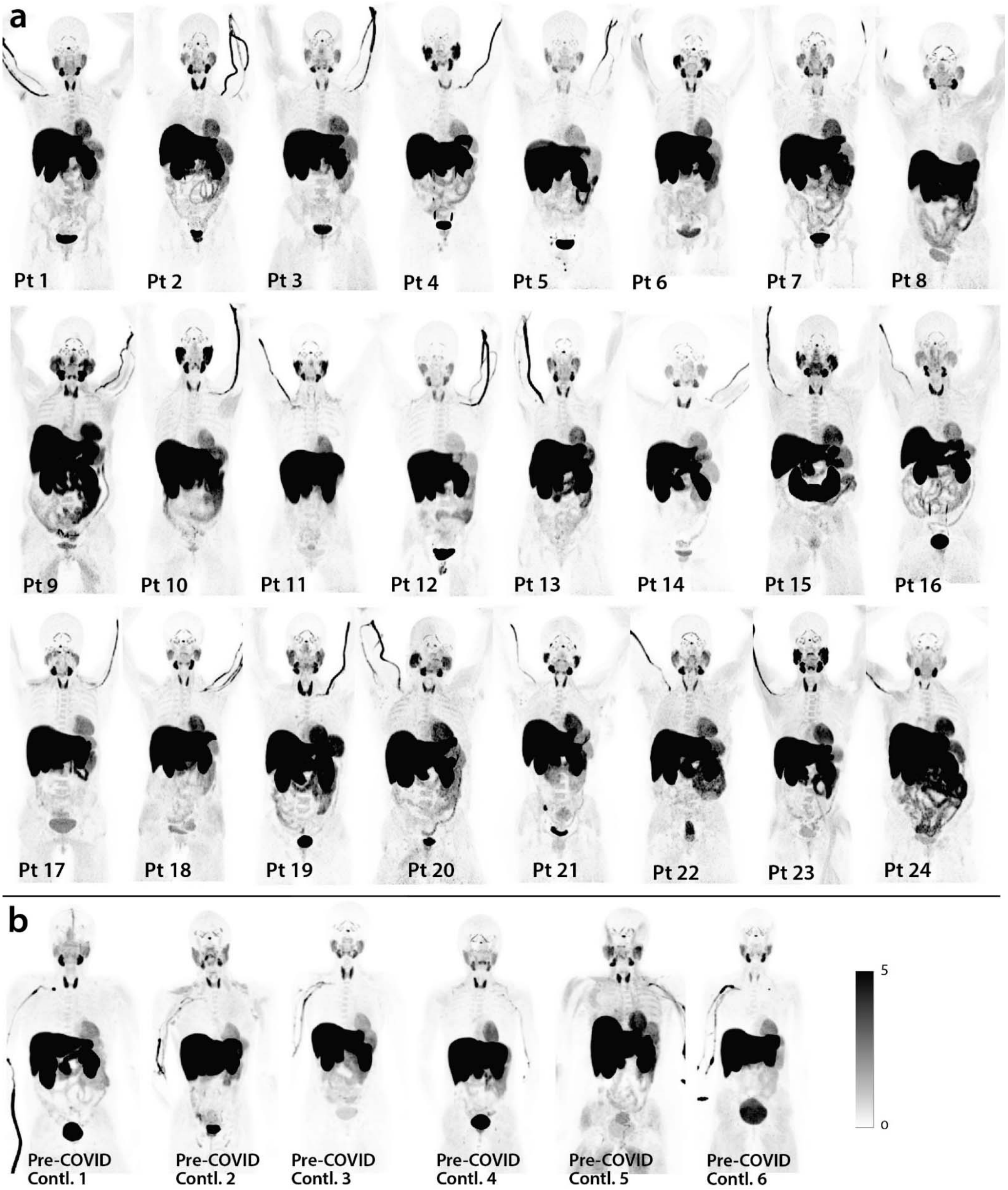

Supplemental Figure 1. Maximum intensity projections (MPI; coronal views of 3-dimesional reconstructions) are shown for all cases and pre-pandemic and contemporary control participants.

medRxiv preprint doi: https://doi.org/10.1101/2023.07.27.23293177; this version posted July 31, 2023. The copyright holder for this preprint
(which was not certified by peer review) is the author/funder, who has granted medRxiv a license to display the preprint in perpetuity.
It is made available under a CC-BY 4.0 International license .

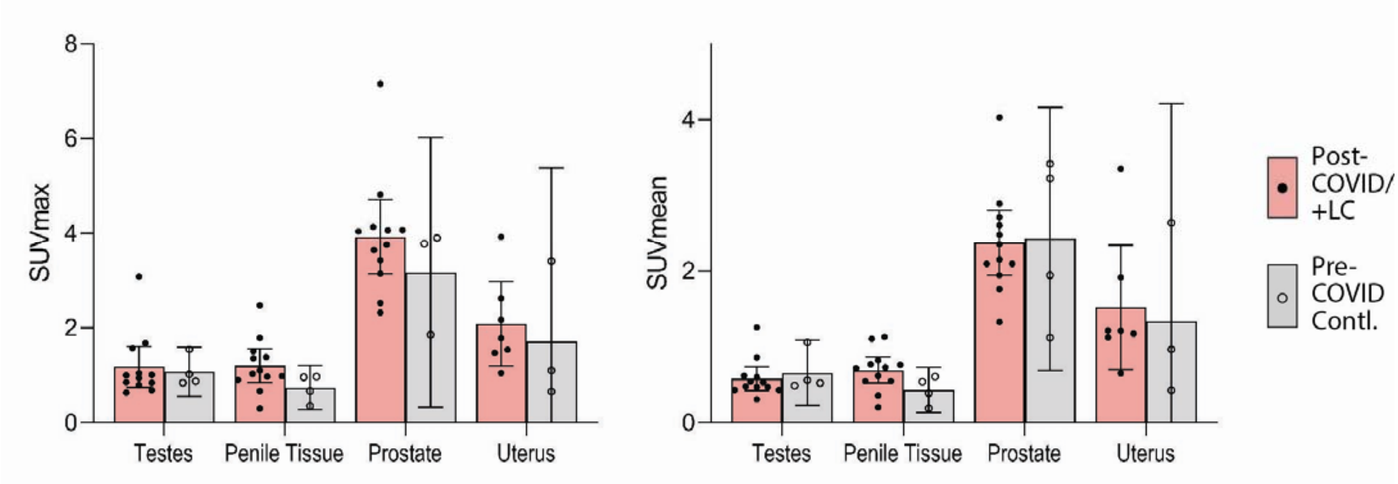

Supplemental Figure 2. [18F]F-AraG SUVmax and SUVmean for reproductive tissues for cases and pre-pandemic controls are shown. Bars represent mean SUVmax and error bars represent 95% confidence interval. No statistical differences were observed between cases and controls for any reproductive tissue ROI. All data points are shown.

medRxiv preprint doi: https://doi.org/10.1101/2023.07.27.23293177; this version posted July 31, 2023. The copyright holder for this preprint (which was not certified by peer review) is the author/funder, who has granted medRxiv a license to display the preprint in perpetuity.
It is made available under a CC-BY 4.0 International license .

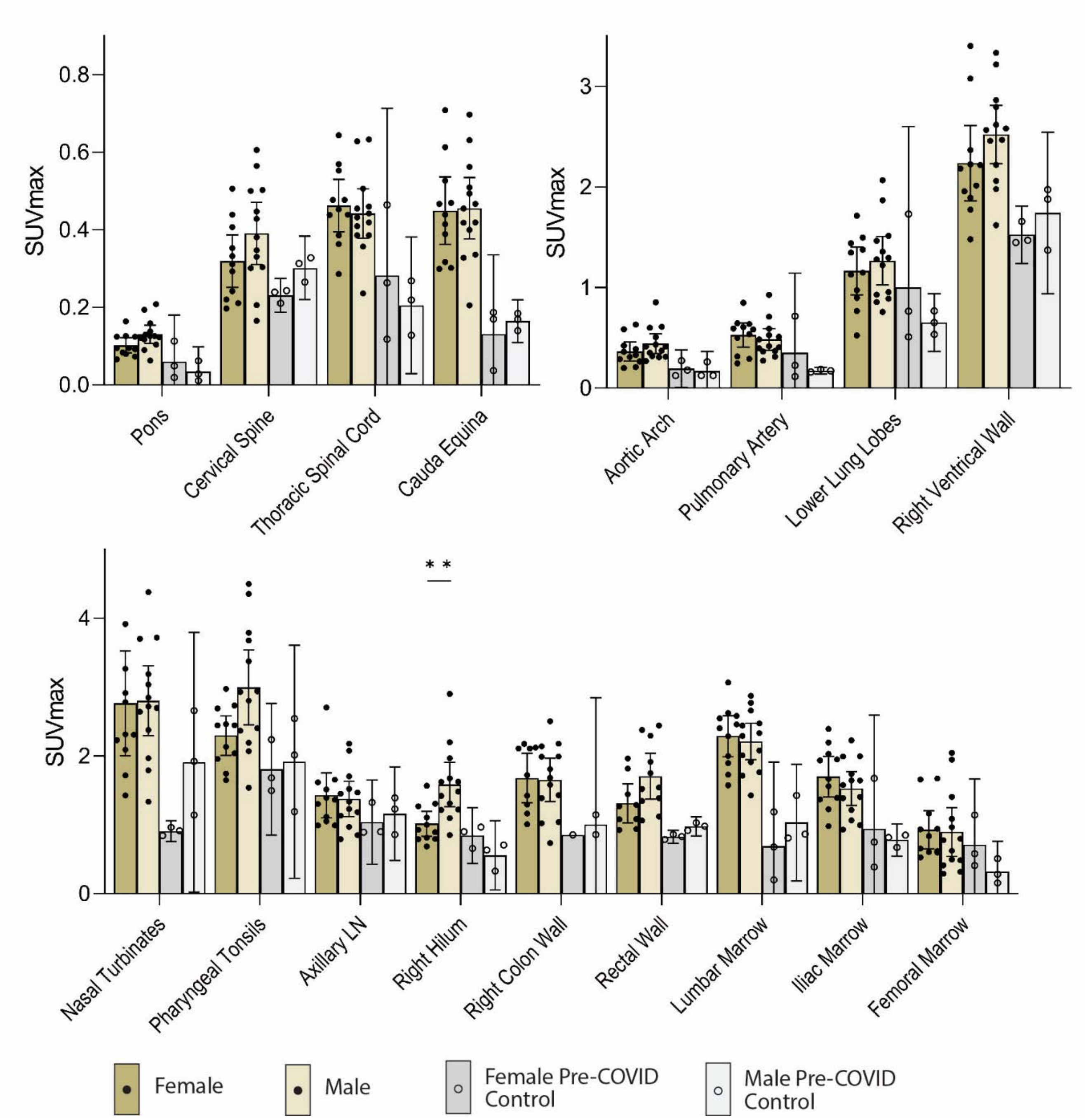

Supplemental Figure 3. Comparisons of [^{18}F]F-AraG maximum standardized uptake values in post-acute COVID cases and pre-pandemic control participants grouped by sex assigned at birth are shown. Bars represent mean SUVmax and error bars represent 95% confidence interval. Adjusted P values <0.05 and <0.001 represented by * and **, respectively from two-sided non-parametric Kruskal–Wallis tests using a Benjamini-Hochberg adjustment for false discovery rates across multiple comparisons (q value = adjusted P). Given lack of power to compare cases versus controls grouped on sex, statistical analyses were only performed between female and male post-acute COVID participants. All data points are shown.

medRxiv preprint doi: https://doi.org/10.1101/2023.07.27.23293177; this version posted July 31, 2023. The copyright holder for this preprint (which was not certified by peer review) is the author/funder, who has granted medRxiv a license to display the preprint in perpetuity.
It is made available under a CC-BY 4.0 International license .

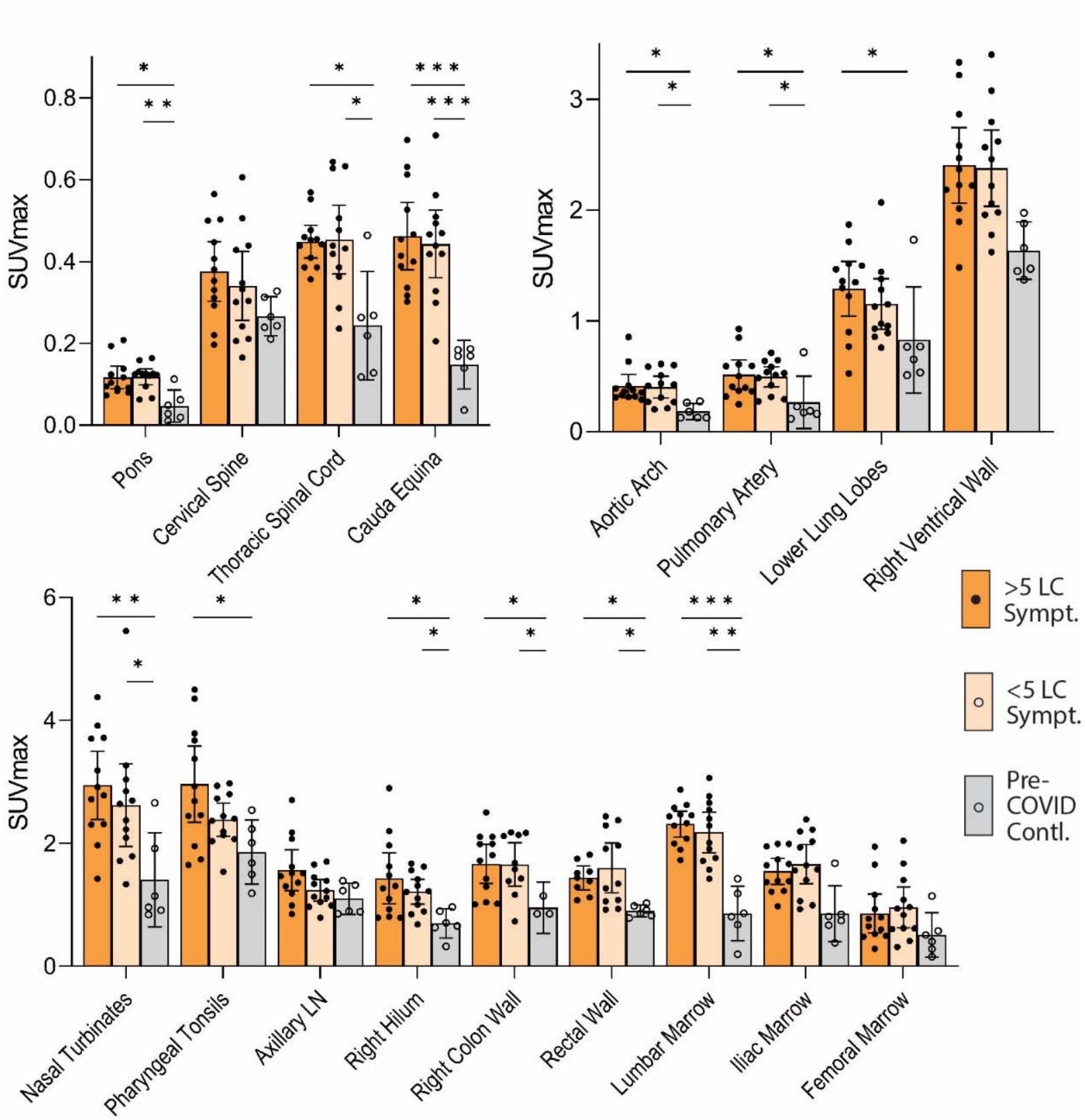

Supplemental Figure 4. Comparisons of [^{18}F]F-AraG maximum standardized uptake values in post-acute COVID cases and pre-pandemic control participants grouped participants with >5 or <u><</u>5 Long COVID symptoms reported at the time of imaging and control volunteers are shown. Bars represent mean SUVmax and error bars represent 95% confidence interval. Adjusted P values <0.05, <0.01 and <0.001 represented by *, **, and *** respectively from two-sided non-parametric Kruskal–Wallis tests using a Benjamini-Hochberg adjustment for false discovery rates across multiple comparisons (q value = adjusted P). All data points are shown.

medRxiv preprint doi: https://doi.org/10.1101/2023.07.27.23293177; this version posted July 31, 2023. The copyright holder for this preprint (which was not certified by peer review) is the author/funder, who has granted medRxiv a license to display the preprint in perpetuity.
It is made available under a CC-BY 4.0 International license .

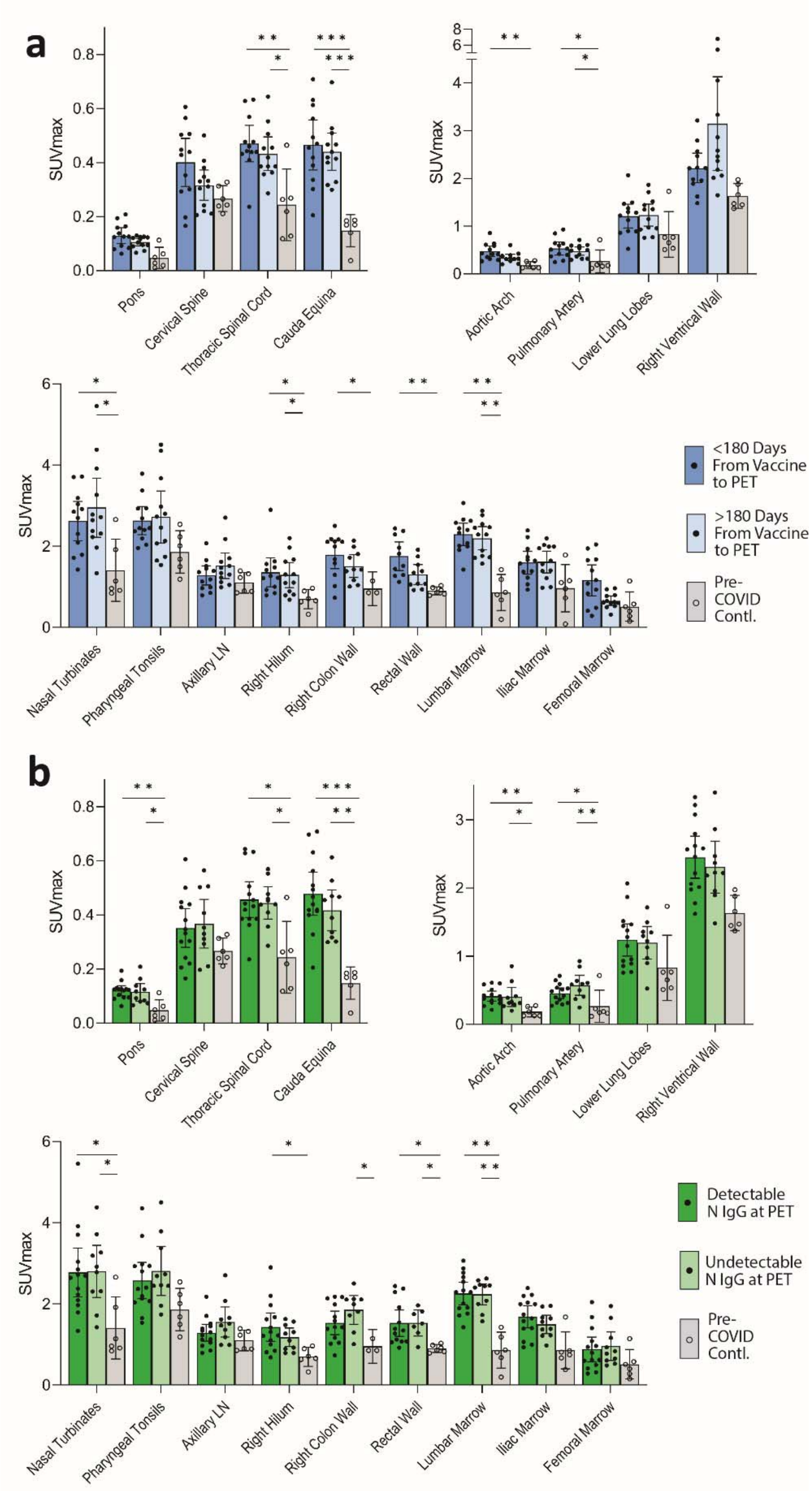

Supplemental Figure 5. Comparisons of [^{18}F]F-AraG maximum standardized uptake values in post-acute COVID cases and pre-pandemic control participants grouped by time from most recent dose of SARS-CoV-2 vaccine and PET imaging and by presence of detectable nucleocapsid (N) IgG detection at the time of imaging. SUVmax values in tissue ROIs in post-acute COVID participants imaged <180 days or >180 days

medRxiv preprint doi: https://doi.org/10.1101/2023.07.27.23293177; this version posted July 31, 2023. The copyright holder for this preprint (which was not certified by peer review) is the author/funder, who has granted medRxiv a license to display the preprint in perpetuity.
It is made available under a CC-BY 4.0 International license .

from the last dose of COVID-19 vaccine and control volunteers are shown in (**a**). SUVmax values in tissue ROIs in post-acute COVID participants with detectable or undetectable SARS-CoV-2 N IgG measures at the time of imaging and control volunteers are shown in (**b**). Bars represent mean SUVmax and error bars represent 95% confidence interval. Adjusted P values <0.05, <0.01 and <0.001 represented by *, **, and *** respectively from two-sided non-parametric Kruskal–Wallis tests using a Benjamini-Hochberg adjustment for false discovery rates across multiple comparisons (q value = adjusted P). All data points are shown.

medRxiv preprint doi: https://doi.org/10.1101/2023.07.27.23293177; this version posted July 31, 2023. The copyright holder for this preprint (which was not certified by peer review) is the author/funder, who has granted medRxiv a license to display the preprint in perpetuity.
It is made available under a CC-BY 4.0 International license .

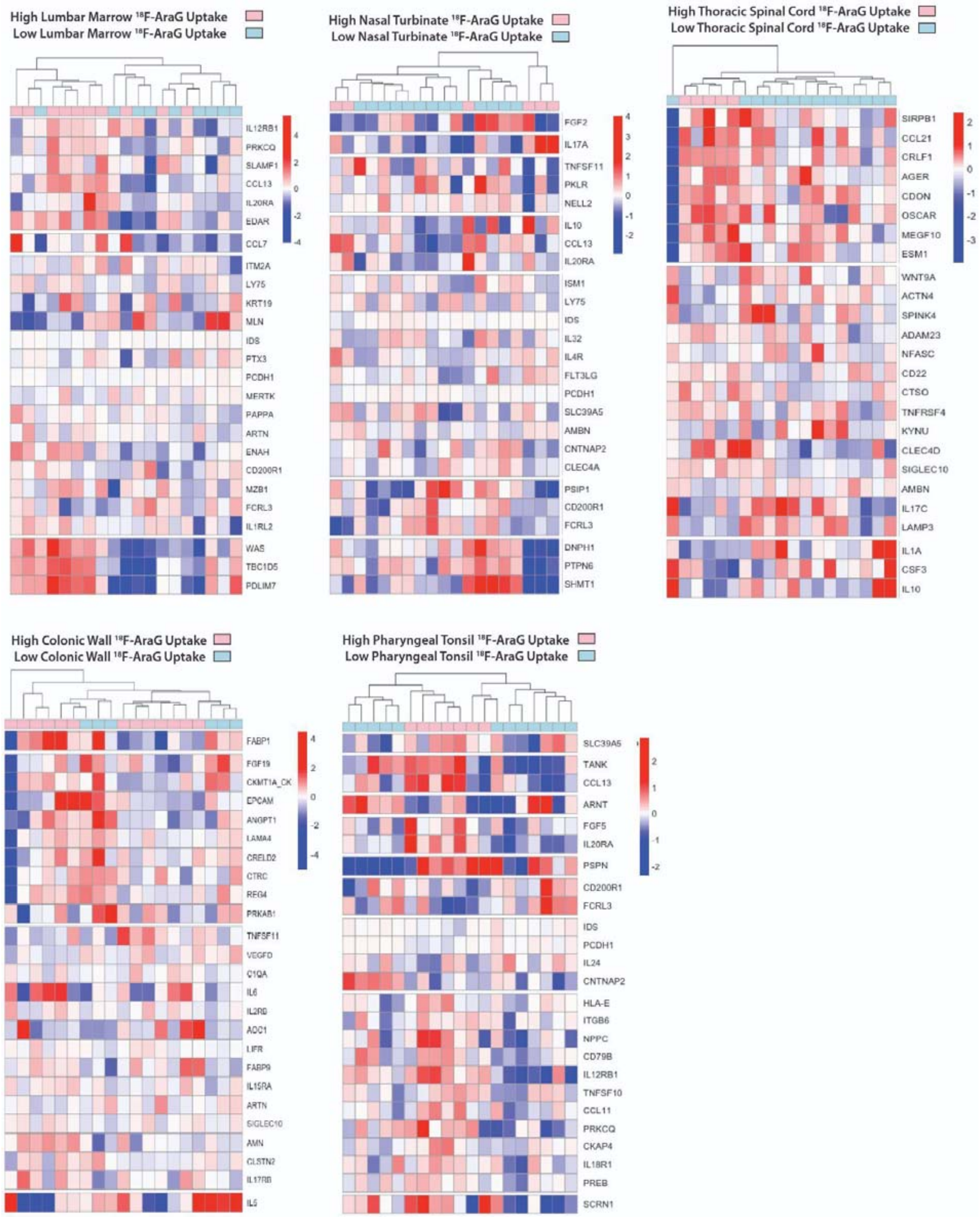

Supplemental Figure 6. Differential plasma protein expression in post-acute COVID participants grouped by high or low [^{18}F]F-AraG uptake in representative tissues. Clustered heat maps of the top 25 differentially expressed plasma proteins from Olink Proximity Extension Assay EXPLORE 384 panel with markers grouped

medRxiv preprint doi: https://doi.org/10.1101/2023.07.27.23293177; this version posted July 31, 2023. The copyright holder for this preprint (which was not certified by peer review) is the author/funder, who has granted medRxiv a license to display the preprint in perpetuity.
It is made available under a CC-BY 4.0 International license .

into k-clusters based on similarity are shown for participants with high or lower PET signal in various tissue ROIs.

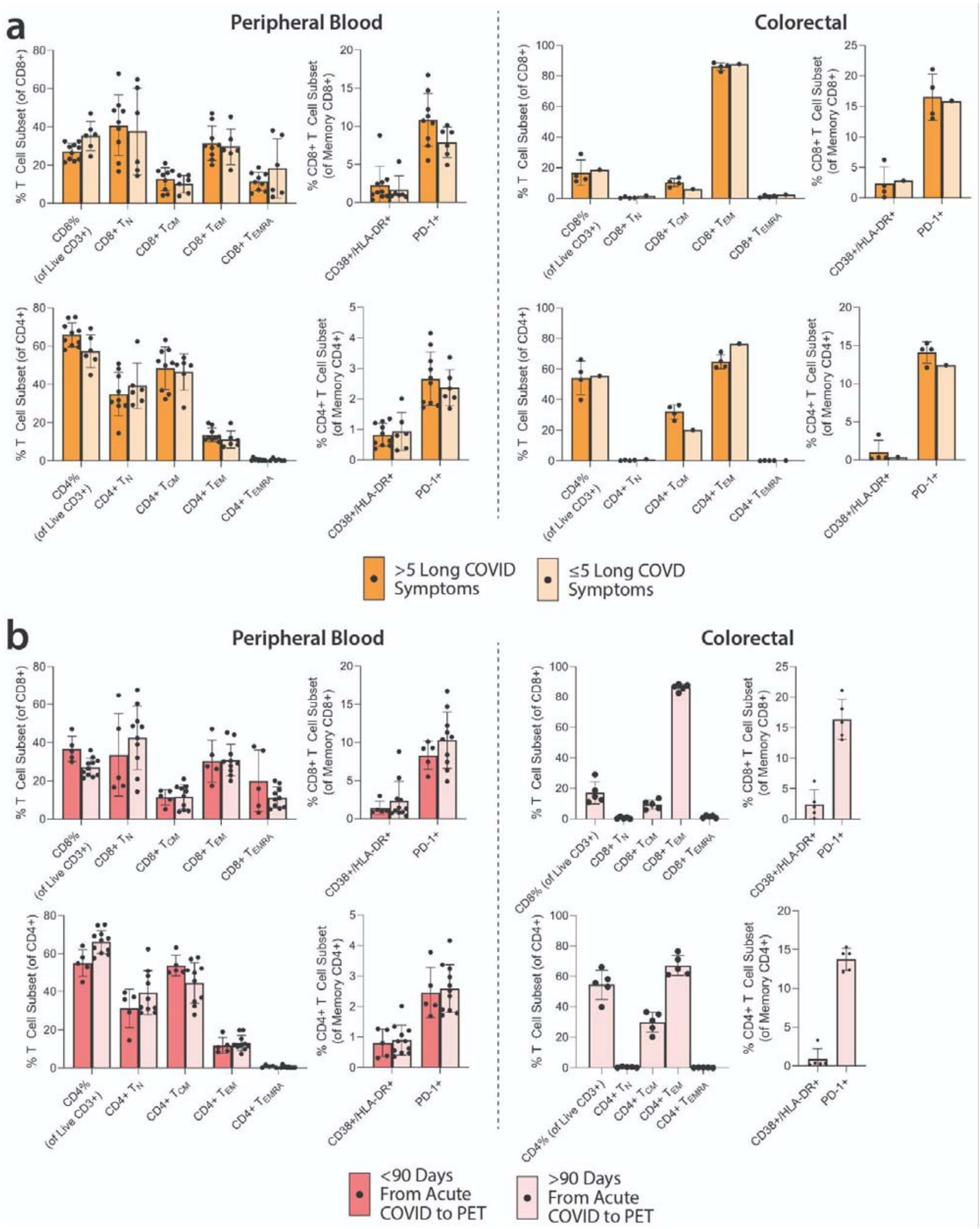

medRxiv preprint doi: https://doi.org/10.1101/2023.07.27.23293177; this version posted July 31, 2023. The copyright holder for this preprint
(which was not certified by peer review) is the author/funder, who has granted medRxiv a license to display the preprint in perpetuity.
It is made available under a CC-BY 4.0 International license .

Supplemental Figure 7. Spectral flow cytometry results for T lymphocyte phenotypes. The frequency of CD8+ and CD4+ T cell subsets (TN = naïve, TCM = central memory, TEM = effector memory, TEMRA = effector memory RA+/terminally differentiated) and frequency of lymphocytes co-expressing activation markers CD38/HLA-DR and immune checkpoint PD-1 from peripheral blood and gut for those with >5 or ≤5 Long COVID symptoms (**a**) and those imaged >90 or <90 days after onset of acute COVID-19 (**b**) are shown. No significant differences between post-acute COVID groups were identified. Bars represent percent of T cells expressing markers of interest and error bars represent 95% confidence intervals.

medRxiv preprint doi: https://doi.org/10.1101/2023.07.27.23293177; this version posted July 31, 2023. The copyright holder for this preprint
(which was not certified by peer review) is the author/funder, who has granted medRxiv a license to display the preprint in perpetuity.
It is made available under a CC-BY 4.0 International license .

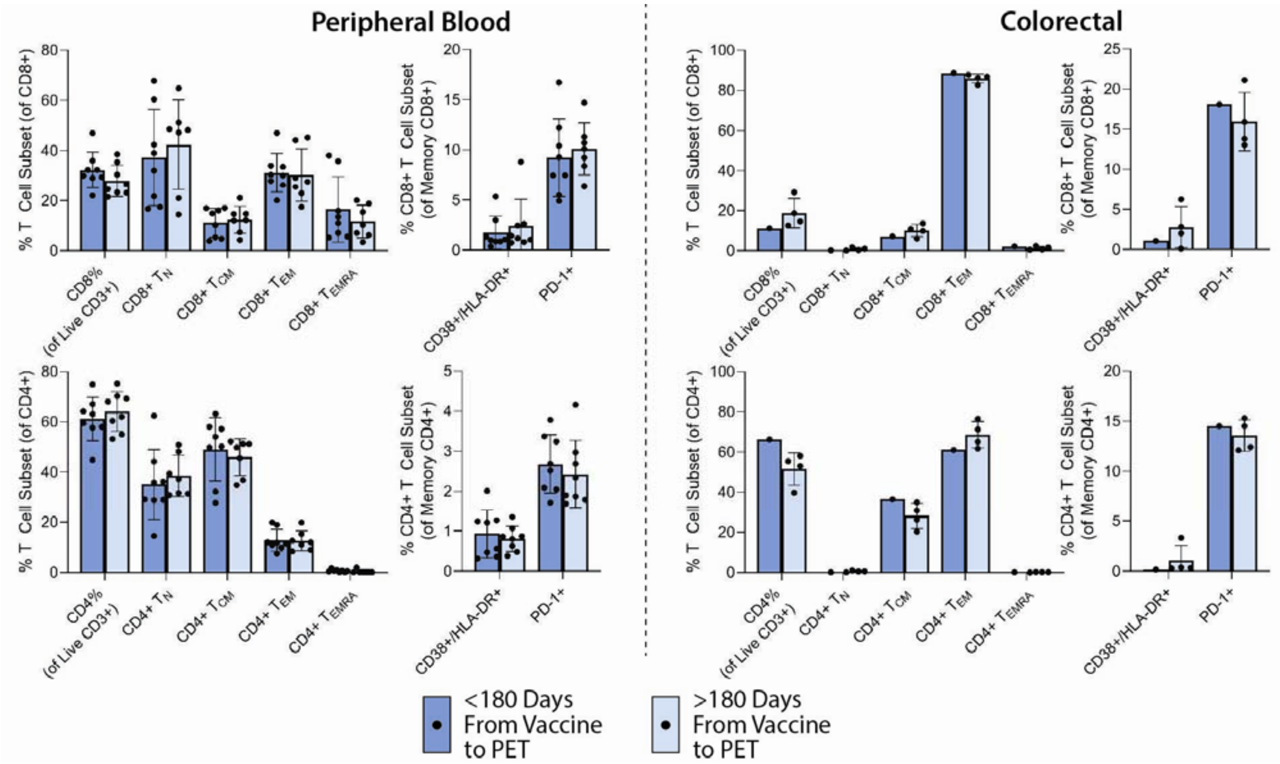

Supplemental Figure 8. Spectral flow cytometry results for T lymphocyte phenotypes grouped by time of PET imaging from last COVID-19 vaccine dose. The frequency of CD8+ and CD4+ T cell subsets (TN = naïve, TCM = central memory, TEM = effector memory, TEMRA = effector memory RA+/terminally differentiated) and frequency of lymphocytes co-expressing activation markers CD38/HLA-DR and immune checkpoint PD-1 from peripheral blood and gut for those who underwent PET imaging <180 or >180 days following the last dose of COVID-19 vaccine are shown. No significant differences between post-acute COVID groups were identified. Bars represent percent of T cells expressing markers of interest and error bars represent 95% confidence intervals.

medRxiv preprint doi: https://doi.org/10.1101/2023.07.27.23293177; this version posted July 31, 2023. The copyright holder for this preprint
(which was not certified by peer review) is the author/funder, who has granted medRxiv a license to display the preprint in perpetuity.
It is made available under a CC-BY 4.0 International license .

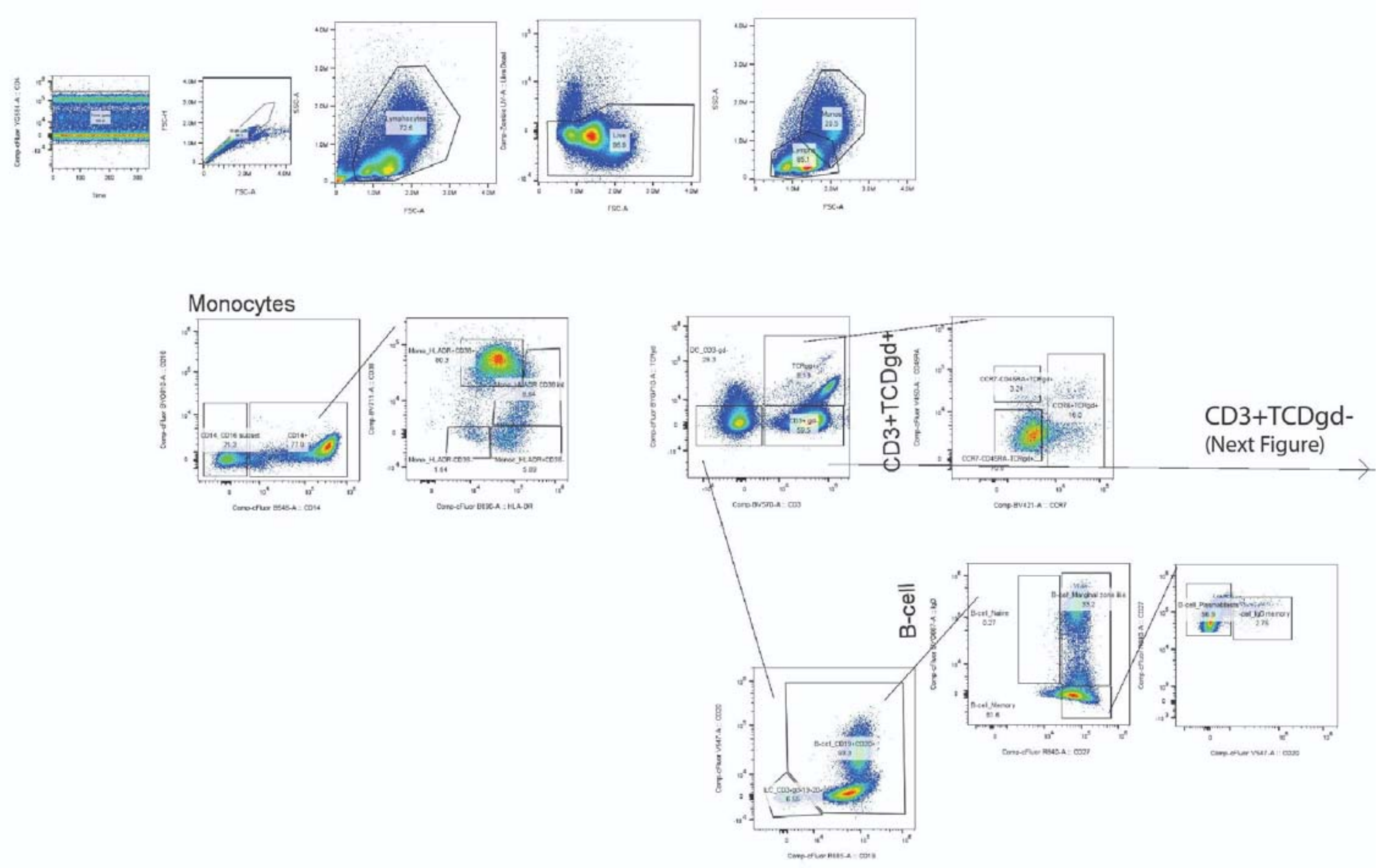

Supplemental Figure 9. Lymphocyte and other mononuclear cell gating strategy (part A).

medRxiv preprint doi: https://doi.org/10.1101/2023.07.27.23293177; this version posted July 31, 2023. The copyright holder for this preprint (which was not certified by peer review) is the author/funder, who has granted medRxiv a license to display the preprint in perpetuity.
It is made available under a CC-BY 4.0 International license .

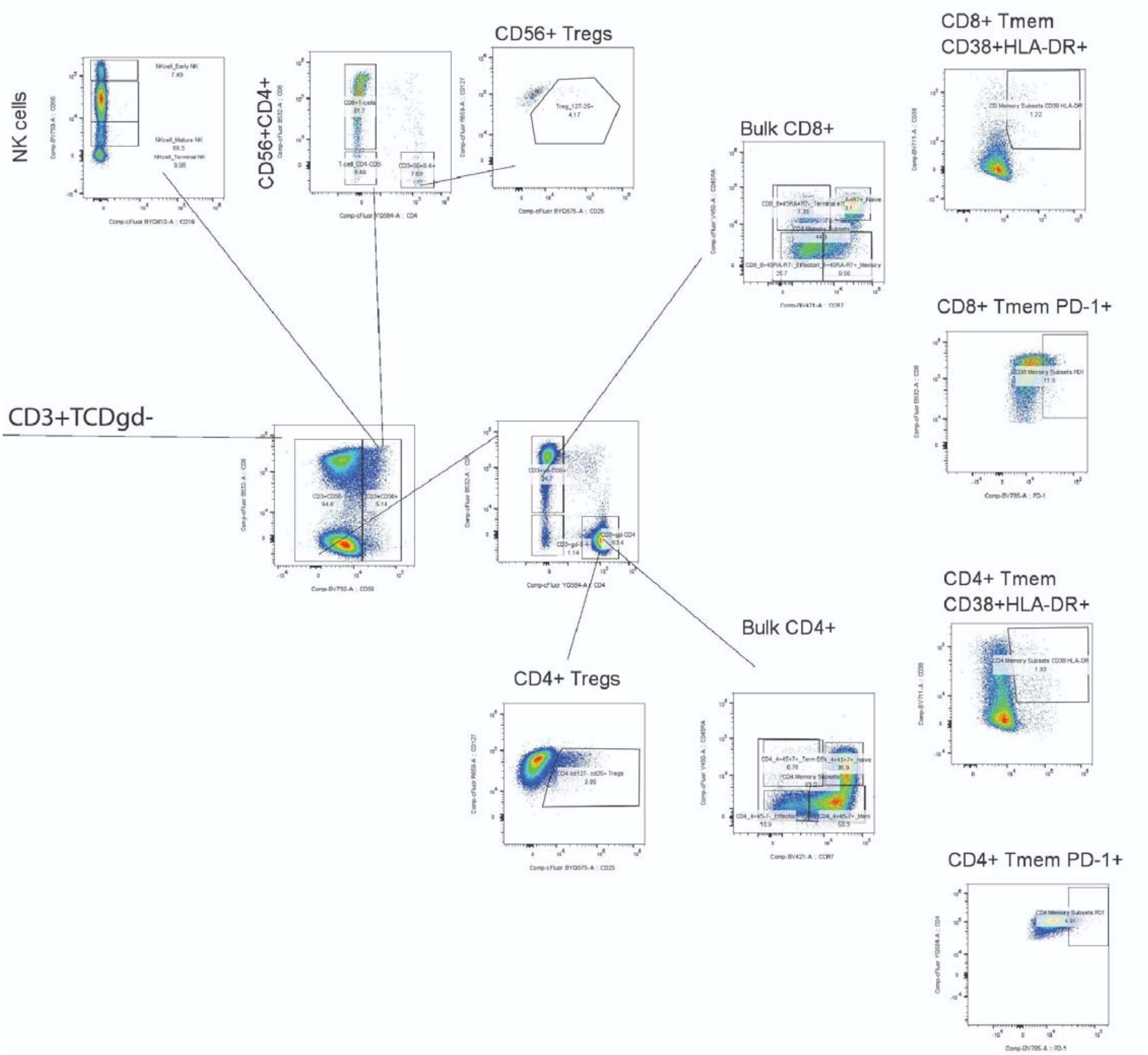

Supplemental Figure 10. Lymphocyte and other mononuclear cell gating strategy (part B).
